# Computational analysis of the MCoTI-II plant defence knottin reveals a novel intermediate conformation that facilitates trypsin binding

**DOI:** 10.1038/srep23174

**Published:** 2016-03-15

**Authors:** Peter M. Jones, Anthony M. George

**Affiliations:** 1 School of Life Sciences, University of Technology Sydney, PO Box 123, Broadway, NSW 2007 Australia.

## Abstract

MCoTI-I and II are plant defence proteins, potent trypsin inhibitors from the bitter gourd Momordica cochinchinensis. They are members of the Knottin Family, which display exceptional stability due to unique topology comprising three interlocked disulfide bridges. Knottins show promise as scaffolds for new drug development. A crystal structure of trypsin-bound MCoTI-II suggested that loop 1, which engages the trypsin active site, would show decreased dynamics in the bound state, an inference at odds with an NMR analysis of MCoTI-I, which revealed increased dynamics of loop 1 in the presence of trypsin. To investigate this question, we performed unrestrained MD simulations of trypsin-bound and free MCoTI-II. This analysis found that loop 1 of MCoTI-II is not more dynamic in the trypsin-bound state than in the free state. However, it revealed an intermediate conformation, transitional between the free and bound MCoTI-II states. The data suggest that MCoTI-II binding involves a process in which initial interaction with trypsin induces transitions between the free and intermediate conformations, and fluctuations between these states account for the increase in dynamics of loop 1 observed for trypsin-bound MCoTI-I. The MD analysis thus revealed new aspects of the inhibitors’ dynamics that may be of utility in drug design.

Knottins are mini-proteins that share a characteristic topology of three disulfide bridges, with one disulfide penetrating through a macrocycle formed by the two other disulfides^1^,^2^. The knottin scaffold is found in almost 30 different protein families across many species. They mediate inhibitory, antimicrobial, insecticidal, cytotoxic, or hormone-like activities and thus are also known as “inhibitor cystine knot” (ICK) proteins^3^. Knottins include marine snail conotoxins, spider toxins, squash inhibitors, agouti-related proteins and plant cyclotides^4^. Cyclotides are plant defence knottins that contain an amide-cyclized backbone^5^,^6^,^7^. The exceptional stability of cyclotides, their high sequence diversity and tolerance to mutation, and their natural roles in high affinity enzyme inhibition has created interest in their use for the design of novel drug leads^8^,^9^,^10^.

MCoTI-I and II are cyclotides isolated from seeds of the spiny bitter cucumber (Momordica cochinchinensis); they are members of the squash trypsin inhibitor family and are potent trypsin inhibitors)^3^,^11^. MCoTI-I and II differ only in changes Q/R and K/R at consecutive residue positions (Fig. 1A). Engineered peptides derived from MCoTI-I have been successful in the inhibition of proteinases of clinical relevance^12^,^13^,^14^.

The solution structure and dynamics of MCoTI-I and II have been investigated using NMR techniques^15^,^16^,^17^,^18^ and recently a crystal structure of trypsin-bound MCoTI-II was reported^19^. An unresolved question arising from these studies concerns the dynamics of the enzyme-bound inhibitor. NMR data for MCoTI-I indicated that regions of the inhibitor, including loop 1 which engages the active site, become more dynamic upon binding to trypsin^18^, whereas the crystal structure of trypsin-bound MCoTI-II suggested the active site loop would become less flexible upon binding.

Atomicscale understanding of dynamic transitions involved in biomolecular recognition is essential to the development of new therapeutics through protein design and engineering. However, characterization of conformational transitions involved in ligand-receptor binding can be difficult to achieve experimentally. X-ray crystallography reveals precise structural snapshots, which can be complemented by NMR experiments that illustrate dynamic changes between conformational substates. Nonetheless, structural interpretation of these motionally averaged, sparse or ambiguous data, to derive the ensemble of states, is complicated. To aid in this, computational sampling procedures, such as MD simulations, can generate possible substate conformations to be compared with experimental data, to derive an atomistic picture of a protein’s dynamics^20^.

Understanding the natural mechanism of action of cyclotides is likely to assist in realising their potential as templates for new drugs. In order to characterise further the interaction between the inhibitor and its natural target enzyme, and to shed light on the apparent discrepancy between the NMR and crystallographic data discussed above, we performed extensive molecular dynamics (MD) simulations of free and trypsin-bound MCoTI-II.

## Results

**Figure 1:**
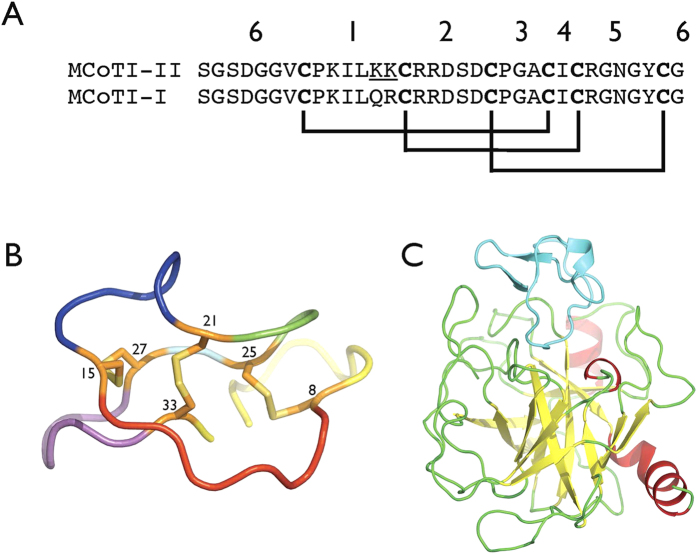
Sequence and structure of MCoTI-II. (**A**) Sequence alignment of MCoTI-I and MCoTI-II. Cysteine residues are shown in bold and disulfide bonds between cysteines are indicated with connecting lines below. Loops spanning successive cysteine residues are numbered 1–6. The two residues that differ between MCoTI-I and MCoTI-II, Lys13 and Lys14 in MCoTI-II in loop 1 are underlined. (**B**) X-ray structure of MCoTI-II used in the simulations. Cysteine residues are numbered with disulfide bonds indicated in stick form with sulphur yellow and carbon orange. Protein backbone is shown in tube representation with loop 1 red, 2 blue, 3 green, 4 cyan, 5 magenta and 6 yellow. The cyclizing peptide bond is omitted to indicate the location of the N- and C-termini. (**C**) X-ray structure of MCoTI-II bound to trypsin used in the simulations^19^ (PDB 4GUX, Chains A and D). Cartoon representation with, for trypsin, β-strands yellow, α-helices red and loop regions green, while MCoTI-II is coloured cyan.

In the following, the residue numbering of MCoTI-II used is as in the crystal structure of trypsin-bound MCoTI-II (PDB 4GUX)^19^, which follows that in the UniProt database entry P82409. MCoTI-I and MCoTI-II are discussed with reference to the six loops which span successive cysteine residues, which form the three disulfide bridges; this is illustrated in Fig. 1A,B.

### Structure and dynamics of free MCoTI-II

**Figure 2:**
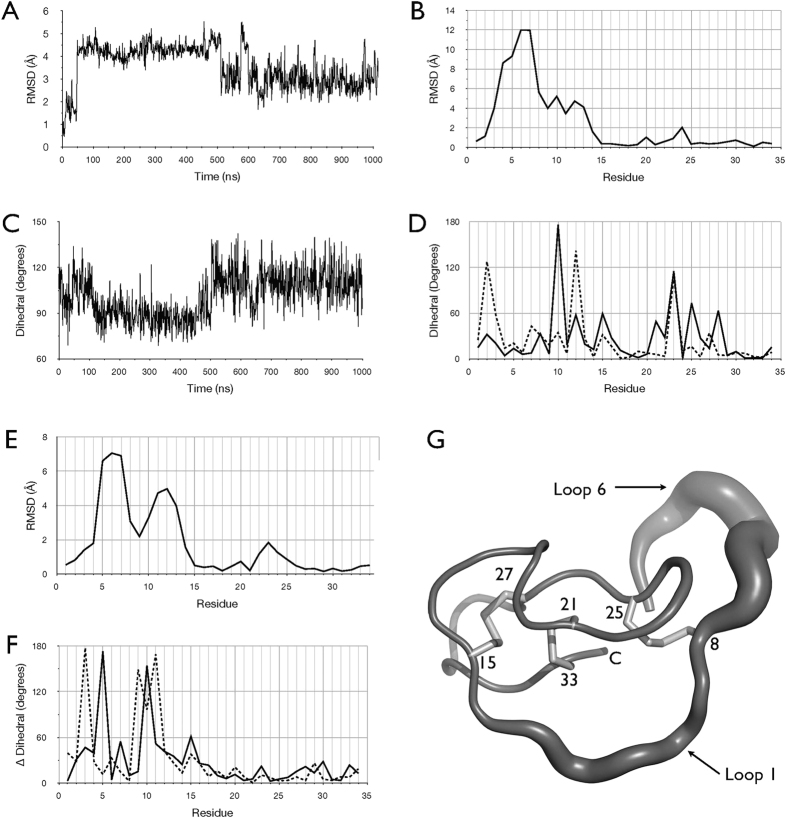
MD simulation of free MCoTI-II. (**A**) Time series of the r.m.s. deviation of Cα atoms from the starting structure, sampled at 1 ns intervals. **(B**) Per residue r.m.s. deviation of Cα atoms between the initial structure and the average simulation structure over the period 50–510 ns. (**C**) Times series of the dihedral angle of the disulfide bond (Cβ-S-S-Cβ) between Cys8 and Cys25, sampled at 1 ns intervals. (**D**) Difference in the backbone Φ (solid line) and Ψ (dashed line) dihedral angles between the starting structure and the average simulation structure over the period 50–510 ns. (**E**) Per residue r.m.s. deviation of Cα atoms between the average simulation structure over the period 50–510 ns and the average structure over the period 510–1000 ns. (**F**) Difference in the backbone Φ (solid line) and Ψ (dashed line) dihedral angles between the average structure over the period 50–510 ns and the average structure over the period 510–1000 ns. (**G**) Residue fluctuations. Structure of MCoTI-II in “sausage” representation, where the thickness of the backbone tube is scaled according to the r.m.s. fluctuations of Cα atoms across the 1 μs simulation. Cysteine residues are indicated by residue number and sidechains shown in stick representations. Loops 1 and 6 are indicated.

A 1.0 μs unrestrained MD simulation was performed of MCoTI-II in a periodic truncated octahedral water cell, using the coordinates of the inhibitor from the crystallographic structure of trypsin-bound MCoTI-II 19 (Fig. 1C). The time series of r.m.s. deviation of Cα atoms from the starting structure (Fig. 2A) reveals that MCoTI-II underwent an abrupt change at around t = 50 ns to a new conformation at which it remained until around t = 510 ns, whereupon it changed abruptly again to a conformation closer to the starting structure.

Insight into the nature of the first significant conformational change that occurs in MCoTI-II is gained from the per residue r.m.s. deviation of Cα atoms between the initial trypsin-bound X-ray structure and the average simulation structure over the period 50–510 ns (Fig. 2B). This reveals that loop 6 and to a lesser extent loop 1 undergo a large conformational change relative to the more stable structural core (loops 2–5), essentially moving about tethers at residues Gly2 and Cys15. Interestingly, this involves a significant displacement of residue Cys8, which pivots via a “crankshaft” motion about the disulfide bond it forms with Cys25, thereby enabling loops 1 and 6 to move in concert. Figure 2C shows how the dihedral angle of the Cys8–Cys25 disulfide bond changes abruptly at t = 50 ns, concomitant with the change in r.m.s. of Cα atoms. This is notable because the three disulfide bonds have been viewed as contributing predominately to structural stability; while the simulation supports this idea for the disulfide bonds Cys15–Cys27 and Cys21–Cys33, it suggests that the Cys8–Cys25 disulfide may also contribute to dynamics and conformational alterations involving the active site binding loop 1 and loop 6.

A plot of the differences in the backbone Φ and Ψ dihedral angles between the starting structure and the average structure over the period 50–500 ns gives further insight into the conformational change that occurs at t = 50 ns (Fig. 2D). Large changes in the backbone dihedrals of the tether points Gly2 and Cys15 indicate they act as hinges for the changes observed in loops 1 and 6, while large changes in the backbone of residues Lys10 and Leu12, which bind directly in the trypsin active site cleft, indicate these residues rapidly adopt a different conformation to the trypsin-bound state. In addition, Cys25, which forms a disulfide bond with Cys8, undergoes a significant backbone change, concomitant with the movement of Cys8. The change in the backbone of Gly23 appears to accommodate changes in the Cys8–Cys25 disulfide bond.

The abrupt change in r.m.s. deviation of Cα atoms from the starting structure that occurs at t = 510 (Fig. 2A) similarly can be characterised by examination of the differences between the average structure over the period 50–510 ns and that for the period 510–1000 ns. The plot of per residue r.m.s. deviation of Cα atoms in Fig. 2E, shows the change at t = 510 ns predominantly involves changes in residues 2 to 15 and to a lesser extent loop 3 (residues 22–24), similar to the regions involved in the change at t = 50 ns. However, the plot of the differences in backbone dihedral angles between the average structures over the intervals 50–510 ns and 511–1000 ns (Fig. 2F) reveals a different pattern to that observed between the starting structure and the average over 50–510 ns (Fig. 2B).

The above analysis indicates that while the conformational changes at t = 50 ns and t = 510 ns involve the same regions, the alterations differ in detail. Most notably, both changes involve a large change in the Φ angle of residue Lys10, a key point of interaction with trypsin in the bound state, and also backbone changes in residue Cys15 at the C-terminal end of loop 1. They differ in that the change at t = 510 ns involves very large backbone changes in loop 6 at residues Ser3 and Gly5, and to a lesser extent at Val7, as well as in the residues either side of Lys10 in loop 1, while the change at t = 50 ns shares none of these dihedral alterations but involves changes in Leu12 in loop 1, as well as in Cys21 and Cys25 at either end of loop 3, in Gly23 in loop 3, and in Arg28 in loop 4.

### Comparison of simulation data with NMR structures of free MCoTI-II

**Figure 3:**
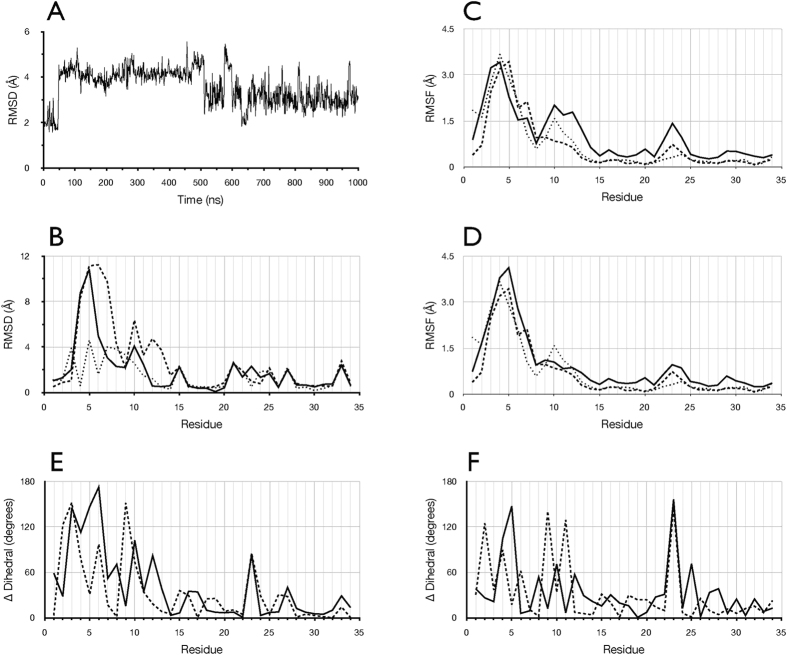
Comparison of free MCoTI-II simulation with NMR structures. (**A**) Times series of the r.m.s. deviation of Cα atoms during the simulation of free MCoTI-II from the NMR lowest energy structure (PDB 1HA9). (**B**) Per residue r.m.s. deviation of Cα atoms between the NMR lowest energy structure and the simulation starting structure (dotted line), the 50–510 ns average structure (dashed line) and the 510–1000 ns average structure (solid line). (**C**) Per residue r.m.s. fluctuations of Cα atoms over the NMR ensembles 1HA9 (dashed line), 1IB9 (dotted line), and the simulation over the 50–510 ns period (solid line). (**D**) Per residue r.m.s. fluctuations of Cα atoms over the NMR ensembles 1HA9 (dashed line), 1IB9 (dotted line), and the simulation over the 510–1000 ns period (solid line). (**E**) Difference in the backbone Φ (solid line) and Ψ (dashed line) dihedral angles between the lowest energy conformer in the NMR structures 1HA9 and 1IB9. (**F**) Difference in the backbone Φ (solid line) and Ψ (dashed line) dihedral angles between the average structure over the period 510–1000 ns and the NMR lowest energy conformer (1IB9).

The NMR solution structure of free MCoTI-II was determined in two independent studies^15^,^16^. To compare these data with our simulation, the time series of the r.m.s deviation of Cα atoms during the free MCoTI-II simulation from the lowest energy NMR conformer (PDB, 1HA9) is plotted in Fig. 3A. This indicates that the conformation over the period 510–1000 ns is closer to the NMR structure than over the period 50–510 ns, but that MCoTI-II does not sample the NMR conformation and indeed remains further removed from the NMR structure than is the starting trypsin-bound conformation.

To compare further the conformation of free MCoTI-II in the simulation and the NMR structure, in Fig. 3B the per residue r.m.s. differences with the NMR structure are plotted for the trypsin-bound starting structure, the average structure over the 50–510 ns period, and the average structure over the 510–1000 ns period. This illustrates that the starting trypsin-bound conformation and the NMR structure differ most in loop 6 and loop 1. Figure 3B also shows that during the simulation, MCoTI-II mostly retains the differences in residues 15–34 between the NMR and the starting structure. To compare dynamics, in Fig. 3C,D, the per residue r.m.s. fluctuations of Cα atoms among the lowest energy conformers determined by NMR are plotted with those calculated from the simulation. Notably, Cα atom fluctuations in loop 1 are greater over the period 50–510 ns than over the 500–1000 ns period during which Cα atom fluctuations are closer to the NMR ensembles.

It should also be noted that the lowest energy conformer from each of the two NMR ensembles, 1HA9 and 1IB9, differ from each other. To illustrate this, Fig. 3E shows the per residue differences in the Φ and Ψ backbone torsion angles between these structures. For comparison, Fig. 3F shows the per residue differences in the Φ and Ψ backbone torsion angles between the average simulation structure over the 510–1000 ns period and the lowest energy NMR conformer 1IB9. This comparison shows that with respect to loop 1, there are significant differences between the NMR structures, and that the 510–1000 ns equilibrated simulation structure does not differ more from one NMR structure than the NMR structures differ from each other. The analysis shown in Fig. 3 indicates that the dynamics and conformation of MCoTI-II over the final 0.5 μs of the simulation is closer to the NMR solution structure than the conformation over the 50–510 ns period. It also shows that dynamics in loop 1 during the simulation are consistent with the variability of loop1 amongst the NMR ensembles.

**Figure 4:**
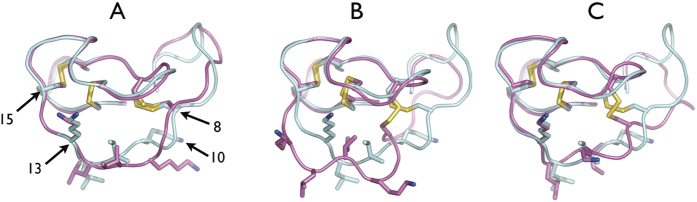
Comparison of the conformation of loop 1 in the free MCoTI-II simulation with the NMR structure. Backbone of MCoTI-II is shown in tube representation, with side chains of cysteines and loop 1 residues 10–13 shown in stick form with nitrogen atoms blue and sulphur atoms yellow. The lowest energy conformer from the NMR ensemble (1HA9) appears in each panel in the same orientation with respect to the viewer, with the backbone tube and carbon atoms coloured cyan; the orientation of the molecule is similar to that in Fig. 1B. The compared structure in each panel is depicted with the backbone tube and carbon atoms coloured magenta. Structures were overlayed using r.m.s. fit of Cα atom coordinates of residues 15 to 21 and 25 to 34. The positions of residues spanning loop 1 in the NMR structure are indicated in panel A. (**A**) Overlay of starting trypsin-bound crystal structure with NMR structure. (**B**) Overlay of 50–510 ns average structure with NMR structure. (**C**) Overlay of 500–1000 ns average structure with NMR structure.

The salient features of the simulation of free MCoTI-II discussed above are illustrated qualitatively in Fig. 4, which depicts structural overlays of the starting conformation, and the average structures over the 50–510 ns and 510–1000 ns periods, with the NMR structure. This shows how the NMR structure of free MCoTI-II is relatively close to that of the trypsin-bound starting structure (Fig. 4A) compared to the average simulation structures over the 50–510 ns (Fig. 4B) and 510–1000 ns periods (Fig. 4C). It also shows how the 510–1000 ns average is closer to the NMR structure than the 50–510 ns average structure, in that in the former the conformation of loop 1 differs substantially only in the deployment of the side chains of residues 10 and 11. The 510–1000 ns average structure also shares with the NMR conformation the relatively compact packing of loop 1 against the structural core of the molecule, in comparison to the 50–510 ns average conformation, and to a lesser extent the trypsin-bound conformation, where loop 1 adopts a more extended conformation (Fig. 4B). In summary, the 510–1000 ns equilibrated simulation free MCoTI-II structure is close to the NMR free structure, and the simulation 50–510 ns intermediate conformation differs from these most significantly in the extended and more accessible conformation of loop 1.

### Structure and dynamics of trypsin-bound MCoTI-II

To investigate MCoTI-II in its enzyme-bound state, seven independent unrestrained MD simulations of the MCoTI-II-trypsin complex in a periodic truncated octahedral water cell were performed, using the coordinates of complex 1 from the crystallographic structure of trypsin-bound MCoTI-II (PDB 4GUX, Chains A and D)^19^. One simulation was run for 340 ns and the other six were run for 125 ns; these are referred to as runs 1–7, respectively. Across the first 125 ns of these seven production runs, the average r.m.s. deviation of all Cα atoms from the starting structure, sampled at 0.1 ns intervals, was 1.26 Å, with the maximum being 2.56 Å; for run 1 the average r.m.s. deviation over 340 ns was 1.30 Å, with the maximum 2.40 Å. Plots of the time series of r.m.s. deviation of all Cα atoms from the starting structure are shown in Fig. S1. These low r.m.s. deviations indicate that the simulations remained stable throughout and that no large global conformational changes occurred in the complex.

**Figure 5:**
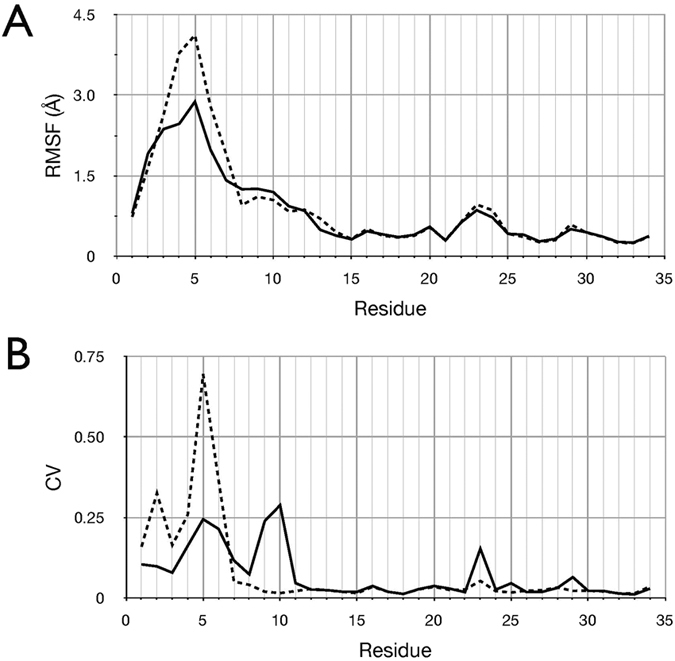
Comparison of residue dynamics between the free and bound state of MCoTI-II. (**A**) Per residue r.m.s. fluctuations of Cα atoms relative to their average position across all trajectory frames for runs 1–7 of the trypsin -bound complex (solid line) and from the simulation of free MCoTI-II in the period 510–1000 ns (dashed line). (**B**) Per residue circular variance of the Φ and Ψ dihedral angle two-dimensional distribution, calculated according to MacArthur and Thornton^21^, from the combined trajectories of the bound state (solid line) and over the period 510–1000 ns from the free MCoTI-II simulation (dashed line).

To examine differences in the dynamics of MCoTI-II between the free and bound forms, r.m.s. fluctuations of Cα atoms relative to their average position across all trajectory frames for runs 1–7 of the trypsin -bound complex are compared to those from the simulation of free MCoTI-II in the period 510–1000 ns (Fig. 5A; see also Fig. 3C). This shows that, while trypsin-bound MCoTI-II exhibited higher Cα atom positional fluctuations for residues 3–6 in loop 6, no appreciable differences were found between the two states for the remainder of the molecule. To compare backbone dynamics further, the circular variance of the backbone Φ and Ψ dihedral angles was calculated across all simulations of the MCoTI-II-trypsin complex (Fig. 5B) and compared to those from the simulation of free MCoTI-II in the period 510–1000 ns^21^. The circular variance is a measure of the range of dihedral angles sampled and is related to the NMR order parameter, which reflects fluctuations of the backbone amide N-H vectors. This analysis indicates that when bound to trypsin, the most significant fluctuations in the MCoTI-II peptide backbone occur in loop 6, in particular residues Gly2 and Gly5. In summary, the simulations do not support the inference from NMR analysis of MCoTI-I that residues Lys10 in loop 1, Arg28 in loop 5, Val7 in loop 6, and Cys15 and Cys33 undergo increased backbone dynamics upon binding to trypsin, relative to the free state.

## Discussion

MD simulations were used to investigate the origin of increased fluctuations of residues in MCoTI-I upon its binding to trypsin, as detected by NMR analysis^18^. The simulations used the crystal structure of the closely related MCoTI-II bound to trypsin as the starting conformation^19^. A 1 μs unrestrained MD simulation of free MCoTI-II in solution, and seven independent unrestrained MD simulations of trypsin-bound MCoTI-II totaling over 1 μs, were performed.

The MD analysis of both free and trypsin-bound MCoTI-II is consistent with both crystallographic and NMR analyses in finding that loop 6 is the most dynamic region of the inhibitor, and that loop 1 is also highly mobile in the free state. The simulation of free MCoTI-II, starting with the trypsin-bound conformation, revealed that the protein rapidly and abruptly changed to an intermediate conformation where it remained for about 0.5 μs, before abruptly transitioning to a conformation that accords closely in terms of structure and dynamics with the free state determined by NMR analysis.

In the simulation of free MCoTI-II, the inhibitor did not adopt the trypsin-bound conformation, even transiently. This is at odds with the NMR solution structure ensembles, where the trypsin-bound conformation was sampled^19^. Nevertheless, as noted previously^17^, in NMR structure determination NOE averaging biases the conformational sampling towards shorter distances, and MD simulations would be expected to provide a more energetically rigorous conformational sampling. The MD analysis thus suggests that free MCoTI-II may more readily or more frequently adopt the 50–510 ns intermediate structure than it will the trypsin bound conformation; however, further replicates to ensure sufficient conformational sampling, together with calculation of the free energies of these states, would be required to support this inference.

The NMR study of free and trypsin-bound MCoTI-I indicated substantially increased backbone dynamics for residues 7, 10, 15, 28 and 33 when the inhibitor bound trypsin^18^ (Equivalent comparative data were not available for residues 1, 3, 4, 5, 8, 9, 11, 14, 22, 24, 25, and 29). Here, MD simulations did not indicate increased fluctuations for MCoTI-II residues 7, 10, 15, 28 and 33 in the trypsin-bound state, relative to the free state (Fig. 5). This agrees with the inference by Daly *et al*^19^ from analysis of the trypsin-bound crystal structure, where it was suggested that the increased fluctuations of the active site lysine 10 observed in the NMR analysis of trypsin-bound MCoTI-I might be due to the inhibitor backbone having been cleaved between residues 10 and 11, consistent with the natural catalytic mechanism of trypsin^19^. Arguing against this, however, when bound [^15^N]-MCoTI-I was removed from trypsin by competition with unlabeled MCoTI-I, NMR analysis indicated it was unaltered^18^. Moreover, this explanation does not account specifically for increased fluctuations in MCoTI-I upon binding to trypsin in residues Arg28 in loop 5, Val7 in loop 6, and Cys15 and Cys33. In addition, it seems at odds with the finding that MCoTI-II is intact in the crystal structure of the trypsin-bound state, although MCoTI-II was found to be cleaved after prolonged incubation with trypsin^19^.

The MD simulations of free MCoTI-II suggest that the conformational change in loops 1 and 6 that occurred at t = 50 ns and t = 510 ns are part of the intrinsic dynamics of the protein and therefore likely to have a functional role. Notably then, the conformational shift at t = 510 ns, as defined by comparing the average structures over the periods 50–500 ns and 510–1000 ns, involves large changes in backbone torsion angles of residues 7, 10, and 15 (Fig. 2F), which correspond to three of the five residues in MCoTI-I detected by NMR analysis to show significantly increased fluctuations in the presence of trypsin. Thus, if during its interaction with trypsin, the conformation of MCoTI-II were to fluctuate between these two conformations, this could give rise to a signal consistent with the NMR order parameters for residues 7, 10, and 15. This interpretation, however, does not account for increased dynamics observed for residues 28 and 33 in the NMR study. Furthermore, in the free MCoTI-II simulation, Leu12 undergoes equally significant backbone dihedral changes to for example residue 7 (Fig. 2F) in the transition between the 50–500 ns and 510–1000 ns average structures, whereas the NMR study did not detect increased dynamics for Leu12 in the presence of trypsin on the ps-ns timescale, although increased dynamics for Leu12 were observed on the μs-ms timescale^18^.

With respect to residue Arg28, it is notable that in order to transition from the 50–510 ns intermediate to the trypsin-bound form, a significant change in the backbone Φ angle of residue 28 must occur (Fig. 2D). However, the transition between these two states cannot account simply for the increased fluctuations observed for residue 28 in the NMR study, because that would also require increased fluctuations for other residues such as Ser2, Leu12 and Gly23 that were not observed in the NMR analysis. Nevertheless, it is also notable that briefly during simulation of free MCoTI-II, a large change in backbone Φ angle of residue 28 did occur, indicating that changes at this point are part of the inhibitor’s natural dynamic repertoire. This was associated with large changes in the backbone dihedral of Gly29 and an overall conformational change in loop 5, which flipped the sidechain of Asn30 outwards away from the core of the molecule. In the simulations of trypsin-bound MCoTI-II, the side chains of both Arg28 and Asn30 were involved in forming hydrogen bonds with a number of residues in trypsin, which dynamically formed, broke and changed partners across the simulations (data not shown). Thus, since loop 5 contains two residues which interact directly with trypsin, it may be that part of the binding process for the inhibitor involves fluctuations in loop 5, similar to those observed here transiently in the simulation of free MCoTI-II, and that these fluctuations account for those observed for residue 28 in the NMR study.

**Figure 6:**
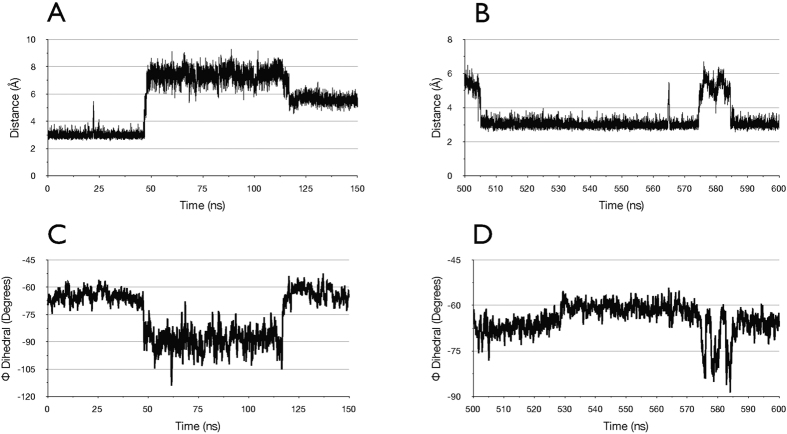
Correlation between changes in the Φ torsion angle of Cys33 and the hydrogen bond with Lys 13 in the free MCoTI-II simulation. (**A**) Distance between amide nitrogen of Cys33 and carbonyl oxygen of Lys13 over the period 0–150 ns. (**B**) Distance between amide nitrogen of Cys33 and carbonyl oxygen of Lys13 over the period 500–600 ns. (**C**) Φ torsion angle of Cys33 over the period 0-150 ns. 10 ps samples, 200 ps moving average. (**D**) Φ torsion angle of Cys33 over the period 500-600 ns. 10 ps samples, 200 ps moving average.

Finally, with respect to Cys33, it is notable that in the crystal structure of trypsin-bound MCoTI-II, and in the conformation between 510–1000 ns in the simulation of free state of MCoTI-II, as well as in the two NMR structures of free MCoTI-II, a hydrogen bond is formed between the backbone amide nitrogen of Cys33 and the backbone carbonyl of Lys13. However, in the simulation of free MCoTI-II, in the transition from the X-ray structure to the intermediate conformation between 50–500 ns, this hydrogen bond is broken and does not reform until the transition at t = 510 ns to the equilibrated free state. This is illustrated in Fig. 6. Panels A and B show the distance between these atoms over the first 150 ns and during the period 500–600 ns, respectively, while panels C and D show the time series of the backbone Φ angle of residue 33 over these same periods. It is clear from these plots that substantial changes in the Φ angle of residue 33 are correlated with breaking of the hydrogen bond. This correlation can be seen most clearly in the period between 570–590 ns, where MCoTI-II undergoes a transient change back to the intermediate conformation (see also Fig. 2A). Thus, as suggested above, if MCoTI-II fluctuates between the intermediate conformation and the free conformation upon initial binding to trypsin, enhanced fluctuations of the backbone Φ angle of residue 33 could occur due to the breaking and formation of the hydrogen bond with Lys13, thereby explaining the order parameter for this residue derived from the NMR data.

## Conclusions

It is clear that increased backbone amide fluctuations observed in MCoTI-I in the presence of trypsin, observed by NMR analysis, could be due to conformational changes involved in the process of binding and unbinding to trypsin, that become prominent due to the dynamic equilibrium between the free and bound states of the inhibitor when in solution with trypsin at high concentration. It is notable then that the transition to and from the intermediate conformation, observed in our MD simulations of the free state, involved changes in backbone dihedral angles that in 4 of 5 cases correspond to backbone amides observed by NMR analysis to undergo increased fluctuations when the closely related MCoTI-I was bound to trypsin. Also notable is that, while the MD data suggest the intermediate state is part of the inhibitor’s functional conformational ensemble, it is unlikely to occur spontaneously in the free or bound state.

The data overall can be interpreted as indicating that the inhibitor undergoes an initial interaction with trypsin, such that the transition between the free and intermediate states is induced, by way of attaining the trypsin-bound form observed in the crystal structure. Since this putative conformational fluctuation involves loops 1 and 6, the initial binding mode must be such that these residues are free to move, but are so placed as to be accessible to their final binding location. In respect of this idea, it is notable that in the average structure representing the 50–510 ns intermediate conformation, loop 1, which engages directly the trypsin active site cleft, is in a more extended conformation than observed in the conformation over final 0.5 μs of the simulation (Fig. 4). This is consistent with the idea that the transition to the 50–510 ns intermediate conformation facilitates binding to trypsin, since it renders the active site loop 1 more accessible. A rationale for this process may be that it protects loop 1 from indiscriminate cleavage by non-target proteases. Although this explanation does not account for differences between the NMR data and the MD data for residues Leu12 and Arg28, we suggest these deficiencies are not greater than those in the alternate explanation that the data arise from the trypsin-bound inhibitor having been cleaved.

In summary, we propose that fluctuations between the 0.5–1 μs equilibrated state and the intermediate conformation observed in the MD simulations of free MCoTI-II, occur as part of the natural binding process to trypsin, and these, together with transitions between the free and bound states, account for the NMR results reported by Puttamadappa *et al*.^18^ for MCoTI-I. These findings may have relevance to the use of these cyclotides and others as templates in the design of new drugs, since it is likely that if the inhibitor employs a process in order to bind, the structural and dynamic features of this process may need to be retained in order to preserve the high affinity these proteins typically have for their target enzyme. Finally, it is also of wider interest that a natural peptide inhibitor might employ such a process, and this may provide opportunities for alternative strategies to nullify their action.

## Methods

The starting coordinates for MD simulations were from the X-ray structure of trypsin-bound MCoTI-II (PDB 4GUX, chains A and D)^19^. Two starting systems were created: System 1 contained MCoTI-II alone and System 2 contained the trypsin-bound MCoTI-II. In both systems the protein was solvated in a truncated octahedral periodic cell with a minimum of 20 Å between periodic images, and neutralised with a 0.2 M NaCl solution. All histidine residues were neutral and protonated at the ε nitrogen, with the exception of the trypsin active site histidine 60, which was neutral and protonated at the δ nitrogen; all other ionisable residues were in the default ionization state.

### Simulation parameters

MD simulations were performed using NAMD version 2.9 22 with the CHARMM27 force field^23^, including Φ/Ψ cross-term map corrections^24^, and the TIP3P model for water^25^. The SHAKE and SETTLE algorithms were used to constrain the bonds containing hydrogens to equilibrium length^26^. A cutoff of 12 Å (switching function starting at 10 Å) for van der Waals and real space electrostatic interactions was used. The particle-mesh Ewald method^27^ was used to compute long-range electrostatic forces with a grid density of approximately 1/Å^3^. An integration time step of 2 fs was used with a multiple timestepping algorithm; interactions involving covalent bonds and short-range non-bonded interactions were computed every time step, while long-range electrostatic forces were computed every two time steps. Langevin dynamics was utilized to maintain a constant temperature of 310 K with a friction coefficient of 5 ps^−1^ on all non-hydrogen atoms. A Langevin piston was used to control pressure with a target of 1 atm, a decay period of 100 fs and a damping timescale of 50 fs.

### Equilibration

The solvated starting structures were minimized using conjugate gradient minimization to a 0.5 kcal/mol.Å root mean square (r.m.s.) gradient with all protein heavy atoms fixed. Water molecules, NaCl ions, and hydrogens were then further minimized during a 50 ps MD run at 310 K, in which all protein heavy atoms were again fixed. This starting model was then minimized with harmonic positional constraints on the NCαCO backbone. A 100 kcal/mol.Å force constant was used to minimise the system to a 0.5 kcal/mol.Å r.m.s. gradient. The constraints were gradually removed by subsequent minimizations to a 0.1 kcal/mol.Å r.m.s. gradient, scaling the initial force constants by factors of 0.5, 0.15, 0.05, and 0. The minimized structure was then heated from 50 K to 310 K in steps of 25 K using velocity reassignment during a 30 ps MD run.

### Production runs

The equilibrated systems were used for the production runs without restraints. System 1 was run for 500 M integration steps equalling 1.0 μs of real time. System 2 was used in seven production runs, for each of which the system was independently equilibrated; the first of these was run for 170 M steps (340 ns) and the other six for 62.5 M steps (125 ns); these are referred to as runs 1–7 respectively. Coordinates were saved every 10 ps for analysis.

### Analysis

VMD^28^, Xplor-NIH^29^ and Simulaid^30^ were used to prepare the system and analyse MD trajectories. All structural figures were prepared using PyMol (http://www.pymol.org/pymol). For analysis, the average structure over a defined period of the simulation of free MCoTI-II is the conformation with the lowest r.m.s. deviation of Cα atoms from the average coordinates, calculated from all frames within the time period.

## Additional Information

**How to cite this article**: Jones, P. M. and George, A. M. Computational analysis of the MCoTI-II plant defence knottin reveals a novel intermediate conformation that facilitates trypsin binding. *Sci. Rep*. **6**, 23174; doi: 10.1038/srep23174 (2016).

## Supplementary Material

Supplementary Information
