# Supplementary material for: Computational analysis of the MCoTI-II plant defence knottin reveals a novel intermediate conformation that facilitates trypsin binding
Source: Sci Rep. 2016 Mar 15;6:23174. doi: 10.1038/srep23174 (PMC4791599; doi:10.1038/srep23174)
Supplement: Supplementary Information [file srep23174-s1.pdf]

Computational analysis of the MCoTI-II plant defence knottin reveals a novel intermediate conformation that facilitates trypsin binding.

Peter M. Jones and Anthony M. George

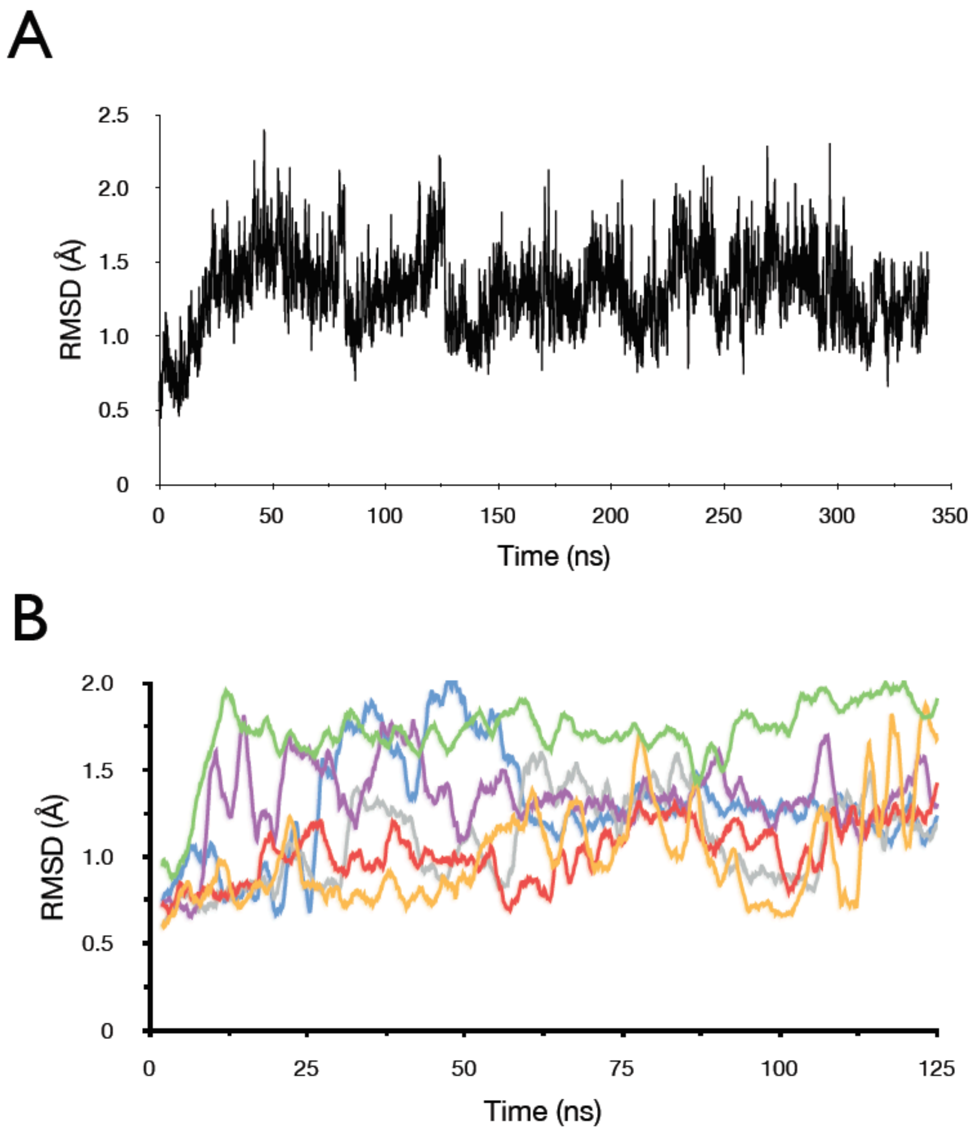

Figure S1. MD simulation of the trypsin-bound MCoTI-II complex.

Time series of the r.m.s. deviation of Cα atoms from the starting structure, sampled at 100 ps intervals. (A) Run 1. (B) Runs 2-7 (samples averaged over a 2 ns window).
